# Initial Establishment of Warning Model for Epidemic Intensity of Norovirus GII Associated with Acute Gastroenteritis in Beijing Based on Synthetic Index Method

**DOI:** 10.3390/v17040473

**Published:** 2025-03-26

**Authors:** Taoli Han, Yan Gao, Shiyao Zhang, Yang Jiao, Jianhong Zhao, Jiaxin Zhao, Yujie Liu, Kuankuan Liu, Pan Lu, Ru Fan, Yuqi Zhang, Xingmei Ren, Mengnan Wang, Zhiyong Gao, Wenjing Li, Beibei Li, Tongyue Su, Lingli Sun

**Affiliations:** 1 Beijing Chaoyang Center for Disease Control and Prevention, Beijing 100021, China; hantaoli0702@163.com (T.H.);; 2 Beijing Center for Disease Control and Prevention, Beijing 100013, China; 3 School of Public Health, Hebei University, Baoding 071000, China

**Keywords:** synthetic index method, norovirus, acute gastroenteritis, wastewater surveillance, average temperatures

## Abstract

At present, there is no research that classifies the epidemic intensity of acute gastroenteritis (AGE) caused by Norovirus (NoV) GII combined cases and environmental surveillance data at the same time. With reference to the experience of the epidemiological-level classification of infectious disease and the actual epidemiological status of NoV AGE in Chaoyang District, Beijing, China, the epidemic intensity of NoV GII was divided into five grades with increasing intensity from grade 1 to grade 5, which corresponds to non-epidemic risk, general risk, moderate risk, high risk, and ultra-high risk, respectively. If the synthetic index of two consecutive monitoring weeks in the epidemic season of 2023–2024 exceeds a certain threshold, an early warning for the corresponding epidemic intensity will be issued and recommendations on the corresponding control measures will be given. This study established and quantified the criteria for the epidemic intensity of AGE caused by NoV GII based on case surveillance data and environmental surveillance data. It provides a reference for other methods to carry out relevant studies in the future. However, mathematical models cannot completely replace skilled experience. Therefore, when making decisions with early warning models in practice, it is necessary to refer to the opinions of professional and experienced experts to avoid the bias of the early warning model from affecting strategy judgment.

## 1. Introduction

Norovirus (NoV) is the primary cause of sporadic cases and outbreaks of acute gastroenteritis (AGE) in all age groups. It has led to about 699 million infected cases [1] and over 200,000 death cases [2] globally. The detection rate of Rotavirus decreased after the introduction and application of the Rotavirus vaccine in China. Since then, NoV has become the most common pathogen [3]. NoV can be classified into 10 genogroups (GI-GX) based on the amino acid sequence diversity of capsid proteins [4], and GI, GII, and GIV can infect humans. In particular, genogroup GII (e.g., GII.4) often leads to global AGE pandemics [4,5,6,7]. 

Timely and accurate surveillance and early warning means are the key to monitoring the AGE and providing timely public health interventions. On the one hand, during the epidemic of COVID-19, wastewater surveillance of SARS-CoV-2 was proved to be an effective and applicable surveillance tool for reflecting the trend of SARS-CoV-2 infections in the population and detected new variants or sub-lineages even earlier than routine clinical surveillance [8,9]. Wastewater-based surveillance has become a useful public health tool for evaluating community infection [10,11]. On the other hand, studies have shown that the average temperature is the main climatic factor influencing NoV outbreaks in humans [12,13]. In addition, the synthetic index method can combine data from various surveillance indicators to establish early warning thresholds for the prevalence level of Norovirus AGE. The moving epidemic method (MEM) has been used as a routine surveillance program to determine the epidemic threshold and intensity level of influenza [14,15]. Therefore, this study combines the data of human health surveillance, wastewater surveillance, and meteorological surveillance in Chaoyang District, Beijing. This study graded the epidemic intensity and provided technical support for the early warning of AGE caused by NoV.

## 2. Materials and Methods

### 2.1. Data Sources

Under the requirements of the *Guidelines on Outbreak Investigation, Prevention, and Control of Norovirus Infection (2015)*, this study investigated outbreaks of AGE from June 2023 to June 2024 in the Chaoyang District of Beijing, China. Schools, kindergartens, and other institutions reported outbreaks to community health centers, and then the latter relayed the information to Chaoyang District CDC. The Chaoyang District CDC conducted investigations within 24 h of receiving a report and collected anal swabs or fecal specimens from cases for pathogen testing within 48 h of initiating the investigation. An AGE outbreak surveillance network has been established in Beijing [16]. In the Chaoyang District CDC, diagnostic testing for AGE outbreaks includes the detection of NoV and other diarrhea viruses (Sapoviruses, Astroviruses, enteric Adenoviruses, and Rotaviruses). A NoV outbreak was confirmed when two or more AGE cases tested positive for NoV by real-time polymerase chain reaction (PCR) testing.

With reference to the requirements of the *National Viral Diarrhea Surveillance Program (2021)* [17], sentinel hospitals, Chuiyangliu Hospital affiliated Tsinghua University and Beijing Children’s Hospital, Capital Medical University, collect at least 10 intestinal outpatient diarrhea cases per month and send to the Chaoyang District CDC for testing of diarrheal viruses such as NoV.

Based on the wastewater-based surveillance network of SARS-CoV-2 by the *Urban Wastewater Surveillance Program of SARS-CoV-2 (2023)*, the copy numbers (copies/mL) of Norovirus GII in wastewater from the wastewater treatment plant in Chaoyang District, Beijing, from June 2023 to June 2024, were collected and detected. The standard curve is plotted with Ct*_GII_* as the vertical coordinate and Lg_(*GII copies/mL*)_ as the horizontal coordinate. After meeting the conditions of the coefficient of determination (*R*^2^) > 0.99 and amplification efficiency (E%) between 90% and 110%, then the number of copies of NoV GII (copies/mL) in the wastewater can be calculated by substituting the *Ct* value of NoV GII into the standard curve equation.

The test results of all the above samples have been reviewed for accuracy by the Beijing CDC.

Daily average temperature data for 2023 to 2024 were obtained from the website of the National Centers for Environmental Information (NCEI) (https://www.ncei.noaa.gov/data/global-summary-of-the-day/archive/ (accessed on 11 November 2024)) from the U.S. National Oceanic and Atmospheric Administration (NOAA). The degrees Fahrenheit were converted to degrees Celsius of daily average temperature for calculation and establishment of the database of weekly average temperatures.

### 2.2. Research Method

Surveillance indicators including routine and indirect surveillance indicators were selected to reflect the prevalence of NoV infection in the population. The MEM program includes three steps: (1) Define the epidemic period of surveillance data. (2) Determine the thresholds of the epidemic and the intensity. (3) Assess the model performance. Further details are available in the study [18]. All original surveillance data were standardized and assigned different weights, and then the Synthetic index (SI) was obtained. The results of laboratory confirmation showed that 89.37% (496/555) [19] of the NoV-associated AGE outbreaks in this laboratory from 2014 to 2023 were caused by GII, and the main pathogen of AGE was still dominated by NoV GII over the years [19,20]. Finally, this study established a hierarchy of prevalence levels of AGE caused by NoV GII based on the distribution characteristics of the SI.

#### 2.2.1. Surveillance Indicators

**Norovirus GII AGE cases**: cases with ≥3 times within 24 h and changes in fecal character, and/or vomit are diagnosed as NoV GII by laboratory testing.

**Norovirus GII weekly positive detection rate of sentinel surveillance (%)**: the number of specimens positive for NoV GII in sentinel hospitals per week divided by the total number of specimens for testing in sentinel hospitals in the same week (×100%).

**Number of weekly outbreaks of Norovirus GII:** the number of outbreaks caused by NoV GII in outbreak surveillance every week.

**Norovirus GII weekly concentration in urban wastewater (copies/mL):** arithmetic mean of concentrations (copies/mL) of weekly NoV GII from urban wastewater of eight wastewater treatment plants (WWTPs) in Chaoyang District, Beijing, from 1 June 2023 to 30 June 2024. 

**Weekly average temperature (°C)**: arithmetic mean of daily average temperatures in degrees Celsius per week. 

#### 2.2.2. Normalized Threshold of Surveillance Indicators

**Normalized threshold for number of weekly outbreaks of Norovirus GII:** With reference to literature reports and expert assessments, 60% of the maximum value of the number of weekly outbreaks of NoV GII in outbreak surveillance in Chaoyang District, Beijing, from 2023 to 2024, was used as the threshold for determining the epidemiological and non-epidemiological periods of AGE. It was determined as the normalized threshold for the number of weekly outbreaks of NoV GII in the outbreak surveillance.

**Normalized threshold for Norovirus GII weekly positive detection rate of sentinel surveillance (%):** Sentinel surveillance was categorized into the epidemic and non-epidemic periods based on the established normalized threshold for the number of weekly outbreaks of NoV GII. The arithmetic mean of the weekly positive detection rate (%) for sentinel surveillance of NoV GII during the epidemic period was used as the normalized threshold.

**Normalized threshold for Norovirus GII weekly concentration of urban wastewater (copies/mL):** Wastewater surveillance was divided into the epidemic and non-epidemic periods using the identified normalized threshold for the number of weekly outbreaks of NoV GII. The arithmetic mean of the average weekly detection concentrations (copies/mL) of NoV GII in urban wastewater during the epidemic period was used as the normalized threshold. The GII concentration weekly in wastewater was normalized according to the Z-Score Normalization formula: z = (x − μ)/σ (z is the normalized value, μ is the mean, and σ is the standard deviation).

**Normalized threshold weekly average temperature (°C)**: Temperature surveillance was divided into the epidemic and non-epidemic periods based on the identified normalized threshold for the number of weekly outbreaks of NoV GII. The arithmetic mean of the average weekly temperature (°C) in Chaoyang District during the epidemic period was used as the normalized threshold.

#### 2.2.3. Determination of Synthetic Index Classification

The database was established by word processing system (WPS). The data were also screened and analyzed. Statistical indicators such as mean, median, and percentile were used as the basis for the classification of synthetic index (SI).

#### 2.2.4. Calculation of Synthetic Index

The weighting coefficients of the surveillance indicators were determined with reference to literature reports [14] and expert assessments, and the weekly synthetic index was calculated by the formula of SI = *∑W_i_ Y_ij_* = *∑(W_i_X_ij_/M_i_)*. Where SI refers to the synthetic index, *W_i_* represents the weighting coefficient of the indicator *i* (*i =* 1, 2, 3, 4), *Y_ij_* refers to the relative value of the indicator *i* in week *j*, *X_ij_* is the measured value of the indicator *i* in week *j*, and *M_i_* is the normalized threshold of the indicator *i*.

## 3. Results and Discussion

### 3.1. The Result of Normalized Threshold for Surveillance Indicators

The maximum number of weekly outbreaks of NoV GII in Chaoyang District from June 2023 to June 2024 was five. Therefore, the normalized threshold for weekly outbreaks of NoV GII was three, which was a criterion to distinguish between epidemic and non-epidemic periods. We calculate the threshold of other indicators during the epidemic period in this study accordingly. The normalized threshold for the NoV GII weekly positive detection rate of sentinel surveillance (%) during the epidemic period was 28.63%. The normalized threshold for the NoV GII weekly concentration of urban wastewater (copies/mL) was 193.18 copies/mL. The normalized threshold weekly average temperature (°C) was 15.34 °C. The four seasons of the disease were selected as the basis for SI studies for both epidemic and non-epidemic periods. However, the AGE potentially has been affected by prevention and control measures of COVID-19 in recent years, and it does not reflect the actual prevalence level of GII infection. This study temporarily estimated the threshold using the data of one epidemic season. In the future, the data will be accumulated, and the study period will be extended appropriately to more accurately predict the prevalence grade of AGE caused by NoV GII.

### 3.2. Assigning Weight Coefficients to Monitor Indicators and Calculating Synthetic Index

**Figure 1:**
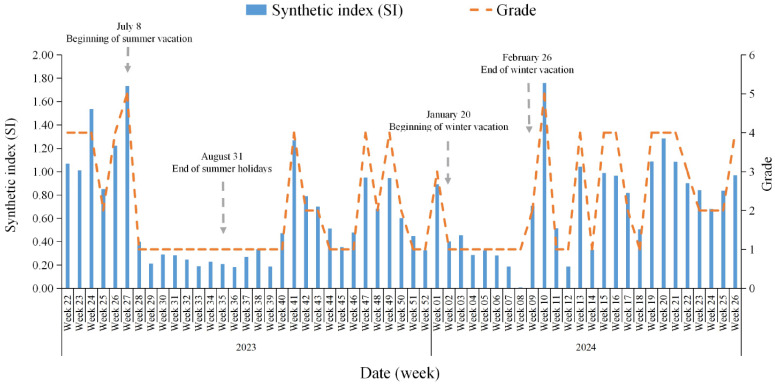
The Trends in Synthetic Index and Epidemic Intensity of Norovirus GII Infections in Chaoyang District, Beijing, China, 2023–2024. Note: 1. Outbreak surveillance: Simultaneous detection of GI and GII in the same outbreak in weeks 3 (GII, 2/3), 21 (GII, 5/6), and 24 (GII, 8/9) of 2024, only one case of GI infection was detected in each epidemic. No co-infection case of GI and GII was found in the same case. In view of the huge volume of samples tested and the high cost of testing in this region, if NoV was detected in an AGE specimen, we stopped testing for Sapoviruses, Astroviruses, enteric Adenoviruses, and Rotaviruses. Therefore, data on co-infection of NoV with Sapoviruses, Astroviruses, enteric Adenoviruses, and Rotaviruses are unavailable. 2. Sentinel surveillance: Co-infection of NoV and enteric Adenoviruses was detected in samples provided by the Beijing Children’s Hospital, Capital Medical University at weeks 23 (2 cases), 24 (4 cases), 25 (1 case), 30 (1 case), 31 (1 case) of 2023, and 15 (1 case) of 2024, respectively. Co-infection of NoV and Astrovirus at week 20 (2 cases), week 24 (2 cases), and week 27 (1 case) of 2023. Co-infection of NoV and Rotaviruses at week 15 (1 case) and week 19 (2 cases) of 2023. No co-infection of NoV and Sapoviruses, NoV GI and GII, was detected. 3. Sentinel surveillance: A Co-infection case of NoV GI and GII was detected in samples provided by the Chuiyangliu Hospital affiliated Tsinghua University at week 14 of 2024. Co-infection of NoV and Sapoviruses at week 28 (2 cases) of 2023 and week 25 (2 cases) of 2024. No co-infection of NoV and Astroviruses, enteric Adenoviruses, and Rotaviruses were detected. 4. The number of weeks with 0 to 5 AGE outbreaks caused by NoV GII were 25, 13, 5, 7, 3, and 4 weeks, respectively.

Because the weekly number of NoV GII for AGE reflects the risk of crowd-sourced transmission, it has a high sensitivity. Therefore, the weighting factor for the number of outbreaks of NoV GII per week was assigned as 0.4. The sensitivity of diarrhea cases in sentinel hospitals was relatively poor, and the NoV GII weekly positive detection rate of sentinel surveillance (%) was assigned a weight of 0.3. The trends of NoV concentration in urban wastewater, environmental temperature, and NoV GII outbreaks were basically similar, but these two indicators are influenced by various factors. These two indicators are correlated with the occurrence of AGE, not causally. And NoV RNA found in WWTP samples provides a broader picture. Then, the NoV GII weekly concentration of urban wastewater (copies/mL) and the weekly average temperature (°C) were assigned weights coefficients of 0.2 and 0.1, respectively. According to the SI formula, the weekly synthetic index dataset of Chaoyang District for the 22nd week of 2023 to the 26th week of 2024 was calculated to have values ranging from 0.0073 to 1.7593, with a median of 0.5155 (Figure 1 and Supplementary Table S1).

### 3.3. Epidemic Intensity of Norovirus GII for AGE

With reference to the experience of epidemiological-level classification of influenza [14,15,18,21] and the actual epidemiological status of NoV AGE in Chaoyang District, the epidemic intensity of NoV GII was divided into five grades. The intensity was increased from grade 1 to 5, corresponding to non-epidemic risk, general risk, moderate risk, high risk, and ultra-high risk, respectively. The median of the weekly SI of 0.52 was used as a threshold to distinguish between epidemic and non-epidemic periods. Based on the expert assessment and the characteristics of the surveillance data, 0.87, 0.92, and 1.71 were used as the cut-off values for classes 2 to 5, respectively (Table 1). If the synthetic index of two consecutive surveillance weeks in the epidemic season of 2023–2024 exceeds a certain threshold, an early warning for the corresponding epidemic intensity is issued and recommendations on corresponding control measures will be given. It is worth noting that mathematical models applied to epidemiological early warning all rely on historical surveillance data, but they cannot completely replace professional experience. Therefore, when making decisions based on the results of the various early warning models in practice, it is necessary to refer to the opinions of authoritative and experienced experts in the field. We should comprehensively consider various factors to avoid bias in the early warning model from affecting the misjudgment strategy.

### 3.4. Early Warning Classification of Norovirus GII AGE for the First Time with the Data of Cases and Environmental Surveillance

At present, there are few studies that have utilized case surveillance data to classify the epidemic intensity of AGE caused by NoV GII. And there are also no studies that use case and environmental surveillance data to do it. In this study, the epidemic intensity of AGE caused by NoV GII was established and quantified by combining case surveillance data and environmental surveillance data. It provides a reference for other methods to carry out relevant studies in the future.

The case samples and their information were collected by the case surveillance systems from outpatient and inpatient departments of sentinel surveillance hospitals. These reflected the incidence and prevalence of AGE to a certain extent. However, it was extremely easy to overlook patients who were not attending hospitals, such as those purchasing medicines from pharmacies and those with asymptomatic infections. Wastewater surveillance could compensate for potential biases in individual clinical testing and is another means of monitoring disease prevalence [22]. For example, poliovirus wastewater monitoring is used to complement AFP surveillance of the Global Polio Eradication Initiative (GPEI), which has been included in the World Health Organization Guidelines for Environmental Poliovirus Surveillance [23,24,25]. 

On the one hand, the dominant strains in wastewater are consistent with human NoV AGE in temporal distribution [26,27]. On the other hand, based on the flowrate-weighted average virus concentration in Hong Kong, significant positive correlation values were observed between the NoV GII concentration in wastewater with the clinical detection rates in fecal specimens (Spearman’s *r* = 0.88 for GII, *p* < 0.01) [28]. Therefore, NoV GII concentration in wastewater was also included as one of the indicators in this study. The wastewater samples in this study cover populations not only in the Chaoyang District of Beijing but also in other regions, which leads to a bias between wastewater surveillance and population testing results. In other words, they are not an exact match. In view of the fact that the average temperature is the main climatic element influencing human NoV outbreaks [12,13], the average temperature was listed as another one of the indicators. In the end, the above indicators were combined to obtain the grading criteria for the epidemic intensity of AGE caused by NoV GII in Chaoyang District, Beijing. In the future, we will also optimize the indicators on the basis of this study and try to introduce more appropriate indicators that can predict the prevalence level of NoV AGE, such as social and economic indicators. The influence of winter and summer vacations on the prevalence of AGE among primary and middle school students is an example (Figure 1).

There are some limitations in this study. The research content in this study will be extended in the following aspects based on the findings: (1) Expanding the geographic scope of the research. (2) Collecting cumulative longitudinal data. (3) Incorporating diversified indicators such as social and economic factors so that the studies will provide a more robust understanding of NoV outbreaks and a more complete picture of the severity of the epidemic. And they will improve the generalizability and replicability of the findings in the future.

## 4. Conclusions

We have initially established and quantified the criteria for the epidemic intensity of AGE caused by Norovirus GII in Beijing for the first time based on case and environmental surveillance data. It provides data support for the timely issuing of early warning information and taking corresponding prevention and control measures, and also serves as a reference for other methods to carry out related research in the future.

## Figures and Tables

**Table 1:** Classification Thresholds of Epidemic Intensity of Norovirus AGE and its Prevention and Control Suggestions in the Chaoyang District of Beijing.

Grade	Thresholds	Epidemic Intensity	Definition	Prevention and Control Suggestions *
Class 1	SI ≤ 0.52	Non-epidemic risk	The number of reported outbreaks of AGE caused by NoV GII and the positive detection rate of NoV GII in sentinel surveillance are low. The outbreak of AGE caused by NoV GII is less likely to spread.	(1) Maintain indoor air circulation and personal hygiene; (2) Maintain good living habits such as adequate sleep, a healthy diet, and moderate exercise.
Class 2	0.52 < SI ≤ 0.87	General risk	The number of reported outbreaks of AGE caused by NoV GII and the positive detection rate of NoV GII in sentinel surveillance has gradually increased. The outbreak of AGE caused by NoV GII is more likely to occur.	Based on Class 1: (1) Schools, kindergartens, and other collective institutions pay close attention to the registration of absences due to illness, as well as to morning and afternoon checks; (2) Multi-channel education on prevention and control of NoV infection and training on standardized handling of vomit and daily disinfection methods, especially in high-incidence places and collective institutions such as schools and childcare institutions; (3) Carry out health surveillance of staff working in high-prevalence places, such as schools and childcare institutions, to avoid the spread of epidemics; (4) The CDC enhanced NoV infection surveillance for timely detection and identification of mutated strains; (5) The CDC stockpiles personnel and supplies for outbreak response, and medical facilities stockpiles personnel and supplies for norovirus acute gastroenteritis diagnosis and treatment.
Class 3	0.87 < SI ≤ 0.92	Moderate risk	The number of reported outbreaks of AGE caused by NoV GII and the positive detection rate of NoV GII in sentinel surveillance is high. The outbreak of AGE caused by NoV GII is easy to transmit.	Based on Class 2: (1) Please stay away from crowded places; (2) Patients with worsening symptoms such as vomiting and diarrhea should seek early medical attention; (3) Schools, childcare institutions, and other collective institutions should not organize large-scale activities as far as possible; (4) The CDC should work with other multi-departmental organizations such as the education department, to conduct supervision and drills for the prevention and control of NoV outbreaks.
Class 4	0.92 < SI ≤ 1.71	High risk	The number of reported outbreaks of AGE caused by NoV GII and the positive detection rate of NoV GII in sentinel surveillance is higher. The outbreak of AGE caused by NoV GII is greatly vulnerable to transmission.	Based on Class 3: (1) If many cases of vomiting and diarrhea occur in collective institutions, suspension from classes or work is recommended; (2) The medical institutions and research institutes should pay close attention to the mutation and severe cases of NoV infection; (3) Multiple sectors are working together to prevent and control outbreaks, and joint working groups are being formed to respond to outbreaks where necessary.
Class 5	SI > 1.71	Ultra-high risk	The number of reported outbreaks of AGE caused by NoV GII and the positive detection rate of NoV GII in sentinel surveillance is quite high. The outbreak of AGE caused by NoV GII is extremely susceptible to transmission and could potentially disrupt school order, lifestyles, production, etc.	Based on Class 4: (1) According to the actual situation and a comprehensive assessment by experts, mandatory control measures, such as suspension of work and classes and restriction of gatherings, are taken at key places and groups of people.

Note: 1. SI refers to the synthetic index; NoV refers to Norovirus; AGE refers to acute gastroenteritis. “*” indicates that the prevention and control suggestions are mainly proposed with reference to the following documents: (1) *Guidelines on Outbreak Investigation, Prevention and Control of Norovirus Infection (2015)* (https://www.chinacdc.cn/jkyj/crb2/qt/nrbdjxwcy/jswj_nrbd/201511/t20151120_298735.html (accessed on 2 July 2024) and https://www.chinacdc.cn/jkyj/crb2/qt/nrbdjxwcy/jswj_nrbd/201511/W020151120324007040854.pdf (accessed on 2 July 2024)); (2) *Establishment of grading thresholds for epidemic intensity of norovirus acute gastroenteritis in Beijing by synthetic index method* (DOI: 10.3760/cma.j.issn.1673-4092.2019.04.004 and https://rs.yiigle.com/cmaid/1160792 (accessed on 6 January 2025)); (3) *Establishment of the classified evaluation system on the levels of influenza epidemics through a synthetic index method, in Beijing* (DOI:10.3760/cma.j.issn.0254-6450.2018.08.016 and http://chinaepi.icdc.cn/zhlxbx/ch/reader/view_abstract.aspx?file_no=20180816&flag=1 (accessed on 2 February 2025)).

## Data Availability

Due to various unavoidable factors, the actual data involved in this study cannot yet be published. But the process and results of the calculation of the synthetic index method are shown in the “Table S1” based on the normalised data.

## Supplementary Materials

The following supporting information can be downloaded at: https://www.mdpi.com/article/10.3390/v17040473/s1, Table S1: Calculation results of surveillance indicators for Synthetic index method.
