# Supplementary material for: Initial Establishment of Warning Model for Epidemic Intensity of Norovirus GII Associated with Acute Gastroenteritis in Beijing Based on Synthetic Index Method
Source: Viruses. 2025 Mar 26;17(4):473. doi: 10.3390/v17040473 (PMC12031202; doi:10.3390/v17040473)
Supplement: Supplementary file 1 [file viruses-17-00473-s001.zip › viruses-3493671-supplementary.pdf]

Supplementary Table S1. Calculation results of surveillance indicators for Synthetic index method

Time		Normalization				Normalization*weight				Synthetic index (SI)	Grade
		Number Of outbreaks	Positive detection rate	Concentration of urban wastewater	Average temperature	Number of outbreaks * 0.4	Positive detection rate * 0.3	Concentration of urban wastewater * 0.2	Average temperature * 0.1		
	Week 22	0.33	0.00	4.08	1.20	0.13	0.00	0.82	0.12	1.07	4
	Week 23	1.00	0.54	1.52	1.48	0.40	0.16	0.30	0.15	1.01	4
	Week 24	0.67	2.04	2.55	1.49	0.27	0.61	0.51	0.15	1.54	4
	Week 25	0.67	0.32	1.70	1.50	0.27	0.10	0.34	0.15	0.85	2
	Week 26	1.33	0.82	1.31	1.83	0.53	0.25	0.26	0.18	1.22	4
	Week 27	1.00	2.79	1.55	1.84	0.40	0.84	0.31	0.18	1.73	5
	Week 28	0.00	0.27	0.75	1.69	0.00	0.08	0.15	0.17	0.40	1
	Week 29	0.00	0.00	0.27	1.59	0.00	0.00	0.05	0.16	0.21	1
	Week 30	0.00	0.15	0.34	1.78	0.00	0.05	0.07	0.18	0.29	1
	Week 31	0.00	0.25	0.18	1.72	0.00	0.07	0.04	0.17	0.28	1
	Week 32	0.00	0.00	0.32	1.82	0.00	0.00	0.06	0.18	0.25	1
	Week 33	0.00	0.00	0.12	1.67	0.00	0.00	0.02	0.17	0.19	1
	Week 34	0.00	0.00	0.27	1.75	0.00	0.00	0.05	0.18	0.23	1
	Week 35	0.00	0.00	0.30	1.49	0.00	0.00	0.06	0.15	0.21	1
	Week 36	0.00	0.00	0.16	1.52	0.00	0.00	0.03	0.15	0.18	1
2023	Week 37	0.00	0.00	0.59	1.52	0.00	0.00	0.12	0.15	0.27	1
	Week 38	0.33	0.00	0.29	1.46	0.13	0.00	0.06	0.15	0.34	1
	Week 39	0.00	0.00	0.25	1.39	0.00	0.00	0.05	0.14	0.19	1
	Week 40	0.33	0.00	1.23	0.93	0.13	0.00	0.25	0.09	0.47	1
	Week 41	1.67	0.00	2.45	1.13	0.67	0.00	0.49	0.11	1.27	4
	Week 42	1.33	0.00	0.82	0.94	0.53	0.00	0.16	0.09	0.79	2
	Week 43	0.33	0.00	2.47	0.74	0.13	0.00	0.49	0.07	0.70	2
	Week 44	0.00	0.00	2.12	0.89	0.00	0.00	0.42	0.09	0.51	1
	Week 45	0.00	0.00	1.44	0.66	0.00	0.00	0.29	0.07	0.35	1
	Week 46	0.00	0.00	2.48	-0.17	0.00	0.00	0.50	-0.02	0.48	1
	Week 47	0.67	0.00	3.35	0.12	0.27	0.00	0.67	0.01	0.95	4
	Week 48	0.00	0.00	3.38	0.07	0.00	0.00	0.68	0.01	0.68	2
	Week 49	0.33	0.00	4.19	-0.25	0.13	0.00	0.84	-0.03	0.95	4
	Week 50	0.00	0.00	3.00	0.03	0.00	0.00	0.60	0.00	0.60	2
	Week 51	0.00	0.00	2.64	-0.79	0.00	0.00	0.53	-0.08	0.45	1
	Week 52	0.00	0.00	2.10	-0.94	0.00	0.00	0.42	-0.09	0.33	1

2024	Week 01	0.33	0.00	4.08	-0.55	0.13	0.00	0.82	-0.06	0.89	3
	Week 02	0.33	0.00	1.52	-0.33	0.13	0.00	0.30	-0.03	0.40	1
	Week 03	0.00	0.00	2.55	-0.53	0.00	0.00	0.51	-0.05	0.46	1
	Week 04	0.00	0.00	1.70	-0.54	0.00	0.00	0.34	-0.05	0.29	1
	Week 05	0.33	0.00	1.31	-0.70	0.13	0.00	0.26	-0.07	0.32	1
	Week 06	0.00	0.00	1.55	-0.27	0.00	0.00	0.31	-0.03	0.28	1
	Week 07	0.00	0.00	0.75	0.37	0.00	0.00	0.15	0.04	0.19	1
	Week 08	0.00	0.00	0.27	-0.46	0.00	0.00	0.05	-0.05	0.01	1
	Week 09	1.67	0.00	0.34	-0.27	0.67	0.00	0.07	-0.03	0.71	2
	Week 10	1.67	3.49	0.18	0.08	0.67	1.05	0.04	0.01	1.76	5
	Week 11	1.00	0.00	0.32	0.51	0.40	0.00	0.06	0.05	0.52	1
	Week 12	0.33	0.00	0.12	0.32	0.13	0.00	0.02	0.03	0.19	1
	Week 13	1.33	1.31	0.27	0.64	0.53	0.39	0.05	0.06	1.04	4
	Week 14	0.33	0.18	0.30	0.83	0.13	0.06	0.06	0.08	0.33	1
	Week 15	1.00	1.50	0.16	1.08	0.40	0.45	0.03	0.11	0.99	4
	Week 16	1.00	1.16	0.59	1.00	0.40	0.35	0.12	0.10	0.97	4
	Week 17	0.67	1.31	0.29	1.01	0.27	0.39	0.06	0.10	0.82	2
	Week 18	0.33	0.70	0.25	1.13	0.13	0.21	0.05	0.11	0.51	1
	Week 19	1.00	1.07	1.23	1.19	0.40	0.32	0.25	0.12	1.09	4
	Week 20	1.00	0.87	2.45	1.31	0.40	0.26	0.49	0.13	1.28	4
	Week 21	1.67	0.44	0.82	1.23	0.67	0.13	0.16	0.12	1.08	4
	Week 22	0.67	0.00	2.47	1.41	0.27	0.00	0.49	0.14	0.90	3
	Week 23	0.33	0.44	2.12	1.54	0.13	0.13	0.42	0.15	0.84	2
	Week 24	0.00	0.78	1.44	1.61	0.00	0.23	0.29	0.16	0.68	2
	Week 25	0.00	0.58	2.48	1.67	0.00	0.17	0.50	0.17	0.84	2
	Week 26	0.33	0.00	3.35	1.66	0.13	0.00	0.67	0.17	0.97	4
